# Clinical significance of CD155 expression and correlation with cellular components of tumor microenvironment in gastric adenocarcinoma

**DOI:** 10.3389/fimmu.2023.1173524

**Published:** 2023-06-27

**Authors:** Xue Liu, Chenyang Xu, Tianwei Guo, Shenghua Zhan, Qiuying Quan, Mengsi Li, Ziyi Wang, Xueguang Zhang, Lingchuan Guo, Lei Cao

**Affiliations:** ^1^ Jiangsu Institute of Clinical Immunology, The First Affiliated Hospital of Soochow University, Suzhou, Jiangsu, China; ^2^ Department of Pathology, The First Affiliated Hospital of Soochow University, Suzhou, Jiangsu, China; ^3^ Department of General Surgery, The First Affiliated Hospital of Soochow University, Suzhou, Jiangsu, China; ^4^ Department of Pathology, Changshu Hospital Affiliated to Nanjing University of Chinese Medicine, Changshu, Jiangsu, China; ^5^ Jiangsu Key Laboratory of Clinical Immunology, Soochow University, Suzhou, Jiangsu, China; ^6^ Jiangsu Key Laboratory of Gastrointestinal Tumor Immunology, The First Affiliated Hospital of Soochow University, Suzhou, Jiangsu, China

**Keywords:** CD155, tumor microenvironment, gastric adenocarcinoma, prognosis, tumor immunotherapy

## Abstract

**Introduction:**

CD155 is recently emerging as a promising target in malignancies. However, the relationship between CD155 expression and tumor microenvironment (TME) cell infiltration in gastric adenocarcinoma (GAC) has rarely been clarified.

**Methods:**

We measured CD155 expression in specimens of gastric precancerous disease and GAC by immunohistochemistry. The association of CD155 expression with GAC progression and cells infiltration in TME was evaluated through 268 GAC tissues and public dataset analysis.

**Results:**

We showed that the expression of CD155 was positively correlated with the pathological development of gastric precancerous disease (r = 0.521, *P* < 0.0001). GAC patients with high CD155 expression had a poorer overall survival (*P* = 0.033). Moreover, CD155 expression correlated with aggressive clinicopathological features including tumor volume, tumor stage, lymph node involvement, and cell proliferation (*P <*0.05). Remarkably, CD155 expression positively related to the infiltration of CD68+ macrophages in TME (*P* = 0.011). Meanwhile, the positive correlation was observed between CD155 and CD31 (*P* = 0.026). In addition, patients with high CD155 expression combined with low CD3, CD4, CD8, IL-17, IFN-γ or CD19 expression as well as those with high CD155 and α-SMA expression showed significantly worse overall survival (*P* < 0.05).

**Conclusions:**

CD155 may play a pivotal role in the development of GAC through both immunological and non-immunological mechanisms and be expected to become a novel target of immunotherapy in GAC patients.

## 1 Introduction

Gastric cancer (GC) is the fourth leading cause of cancer-related death worldwide, with gastric adenocarcinoma (GAC) being most common type (1). Immune checkpoint inhibitors, such as monoclonal antibodies against programmed death-1/programmed death-ligand 1 (PD-1/PD-L1), have revolutionized cancer immunotherapy in a variety of malignancies by alleviating immune-escape and promoting autoimmune activation (2–4). However, anti-PD-1/PD-L1 therapies have limited application in GAC due to uncertain efficacy (5–7). Therefore, it is important to explore novel targets improving responsiveness of immunotherapy and prognosis of GAC.

T-cell immunoglobulin and immunoreceptor tyrosine-based inhibitory motif domain (TIGIT), an co-inhibitory checkpoint receptor widely expressing on T and NK cells, is a marker of exhaustion of T cell function (8). Currently, numerous trials of TIGIT-targeted antibodies alone or in combination with anti-PD-1/PD-L1 are in progress, providing therapeutic strategies to enhance anti-tumor immune responses (8, 9). In addition, CD155 is the principal ligand of TIGIT with high affinity interaction than others (10). Patients with high TIGIT and/or CD155-expressing tumor may benefit from TIGIT blockade better. CD155, known as poliovirus receptor or Nectin-like molecule 5, is a glycoprotein of the immunoglobulin superfamily and emerging as a potential target in immunotherapy (11). CD155 can also interact with the co-inhibitory receptor (CD96) and co-stimulatory receptor (CD226) to suppress or activate T/NK cell-mediated immune responses, respectively (12, 13). Meanwhile, CD155 has various biological functions like regulating cell proliferation, migration, adhesion and polarization, and plays an important role in tumor progression (14, 15). Rarely expressed in normal cells, CD155 usually overexpresses in many human tumors (16–24), and is associated with poor prognosis.

Numerous studies demonstrated tumor microenvironment (TME) can serves as novel targets for tumor immunotherapy (25–27). Recently, the interaction between immune checkpoint molecules and TME has attracted increasing attention. It has been reported that CD155/TIGIT signaling can suppress CD8+ T cell activity (28, 29). However, the relationship between CD155 expression and TME cell infiltration in GAC has rarely been clarified.

In this study, we detected CD155 expression in distinct development stages of gastric cancer, including chronic superficial gastritis (CSG), chronic atrophic gastritis (CAG), low-grade intraepithelial neoplasia (LGIN), and high-grade intraepithelial neoplasia (HGIN). We further analyzed the relationship between CD155 expression and tumor progression and the cellular components of TME in GAC.

## 2 Materials and methods

### 2.1 Specimens of patients with precancerous disease

We collected specimens in different pathological stages of the precancerous gastric disease acquired from Pathology of the First Affiliated Hospital of Soochow University from 2016 to 2021, obtaining 20 cases of CSG, CAG, LGIN and HGIN respectively. All specimens were fixed with formalin, embedded in paraffin and diagnosed by two senior pathologists by Hematoxylin-eosin (HE) staining. Then, the samples were cut into 4 µm slides, and performed by immunohistochemistry (IHC) for CD155.

### 2.2 Bioinformatics of correlation of CD155 with tumor progression and immune cells

#### 2.2.1 Expression of CD155 in STAD

TCGA-STAD data set (https://tcga-data.nci.nih.gov/tcga) was used to explore the expression level of CD155 in stomach adenocarcinoma (STAD). According to the corresponding clinical data of 407 STAD patients in the TCGA-STAD data set, we analyzed the relationship between CD155 expression level and clinicopathological characteristics. Receiver operating characteristic (ROC) curve was performed to assess the diagnostic value of CD155 in STAD. In addition, we collected the expression data of CD155 in various molecular subtypes and immune subtypes of STAD from TISDB website (http://cis.hku.hk/TISIDB/index.php).

#### 2.2.2 Functional enrichment analysis

Person correlation analysis was performed to evaluate the correlation between CD155 and other genes using the TCGA-STAD dataset. We selected the top 300 genes with the highest correlation coefficient for further functional analysis. In addition, 10 proteins blinding to CD155 were obtained from the STRING database according to the following criteria: active interaction sources of experiments and low confidence (0.150). These 10 proteins were validated by previous studies. Ultimately, 10 CD155-binding proteins and 300 candidate genes were used for GO and KEGG enrichment analysis by the R package cluster Profiler.

#### 2.2.3 Gene set enrichment analysis

To explore the potential function of CD155, we performed GSEA algorithm to estimate the altered signaling pathways between high and low CD155 expression groups. Hallmark gene sets were acquired from the MSigDB Collection. The analysis was conducted with 5,000 gene set permutations.

#### 2.2.4 Immune cell infiltration

The CIBERSORT algorithm was utilized to estimate the abundance of 22 types of immune cells for each TCGA-STAD sample. The correlation between CD155 expression level and infiltrating immune cells was evaluated by Person correlation analysis.

### 2.3 Specimens of GAC and tissue microarray (TMA) construction

We retrospectively examined specimens of 268 patients with GAC who underwent surgery in 2011. The specific characteristics of the patient, tissue sample acquisition and TMA construction can refer to our previous study (30). We retrieved the clinical data including gender, age, tumor volume, tumor differentiation, tumor stage, tumor depth, lymph node status and metastasis. Survival time was defined as the time from the start of treatment to the date of death or December 2017. It needs to be emphasized that information on overall survival was available for only 198 patients.

### 2.4 IHC

IHC was performed on the TMA sections according to the protocol described previously (31). The information on the primary antibody used in the experiment was described as follows: anti-CD155 (D8A5G, Cell Signaling Technology), anti-Ki67 (8H5, ZSGB-BIO), anti-CD3 (LNl0, ZSGB-BIO), anti-CD4 (UMAB64, ZSGB-BIO), anti-CD8 (EP334, ZSGB-BIO), anti-Foxp3 (1054C, R&D Systems), anti-IL-17 (AF-317-NA, R&D systems), anti-IFN-γ (sc-74108, Santa Cruz Biotechnology), anti-CD19 (EPl69, ZSGB-BIO), anti-CD11c (5D11, ZSGB-BIO), anti-CD56 (UMAB83, ZSGB-BIO), anti-CD68 (KP1, ZSGB-BIO), anti-CD31 (UMAB30, ZSGB-BIO), and anti-α-SMA (1A4, ZSGB-BIO).

### 2.5 Evaluation of IHC staining

All slides were scanned with a Dmetrix image system. The staining results were assessed by two pathologists independently in a blinded manner. The score of CD155 staining was performed by both immunostaining intensity and proportion in diseased gland or cancer cells. The intensity was scored under high magnification (×200) defining as follows: 0, 1+, 2+, and 3+. The percentage of CD155-positive cells among diseased gland or total tumor cells was estimated as four levels: 0 (<5%), 1 (5-25%), 2 (26-50%), 3 (>50%). The immunoreactive score was calculated as the intensity multiplied by the percentage of stained cells. And the sections scoring 0 were regarded as no expression, scoring 1-4 as weak, scoring 5-7 as moderate, scoring 9 as strong. The patients graded as no and weak expression were classified as the low expression group, and others high expression group. The histoscore (H-score), ranging from 0 to 300, was assessed by adding the multiplication of the different staining intensities in 4 gradations with each percentage of positive cells (30).

For molecules (Ki67, CD3, CD4, CD8, IL-17, CD19, CD11c, CD56, CD68, CD31, and α-SMA), the expression level was assessed by the percentage of stained cells in total number of cells, with <10% staining defined as low expression group and ≥10% as high. In the case of Foxp3 and IFN-γ staining, the expression level was evaluated by manually counting the number of stained cells in five randomly selected fields under high magnification (×200), with <50 defined as low expression group and >50 as high.

### 2.6 Statistical analysis

Statistical analysis was performed using R software 3.6.3 and SPSS Statistics 23.0. Chi-square test was used for comparison of various groups. Spearman’s correlation was applied to analyze relationship between variables. ROC curve was designed to assess predictive value of CD155 in diagnosing intraepithelial neoplasia. Kaplan-Meier survival curve and log-rank test were performed to evaluate survival differences. Cox proportional-hazards regression model were conducted to identify independent risk factors. Statistical significance was set at *P <*0.05.

## 3 Results

### 3.1 CD155 expression in different pathological stages of precancerous diseases of the stomach

**Figure 1:**
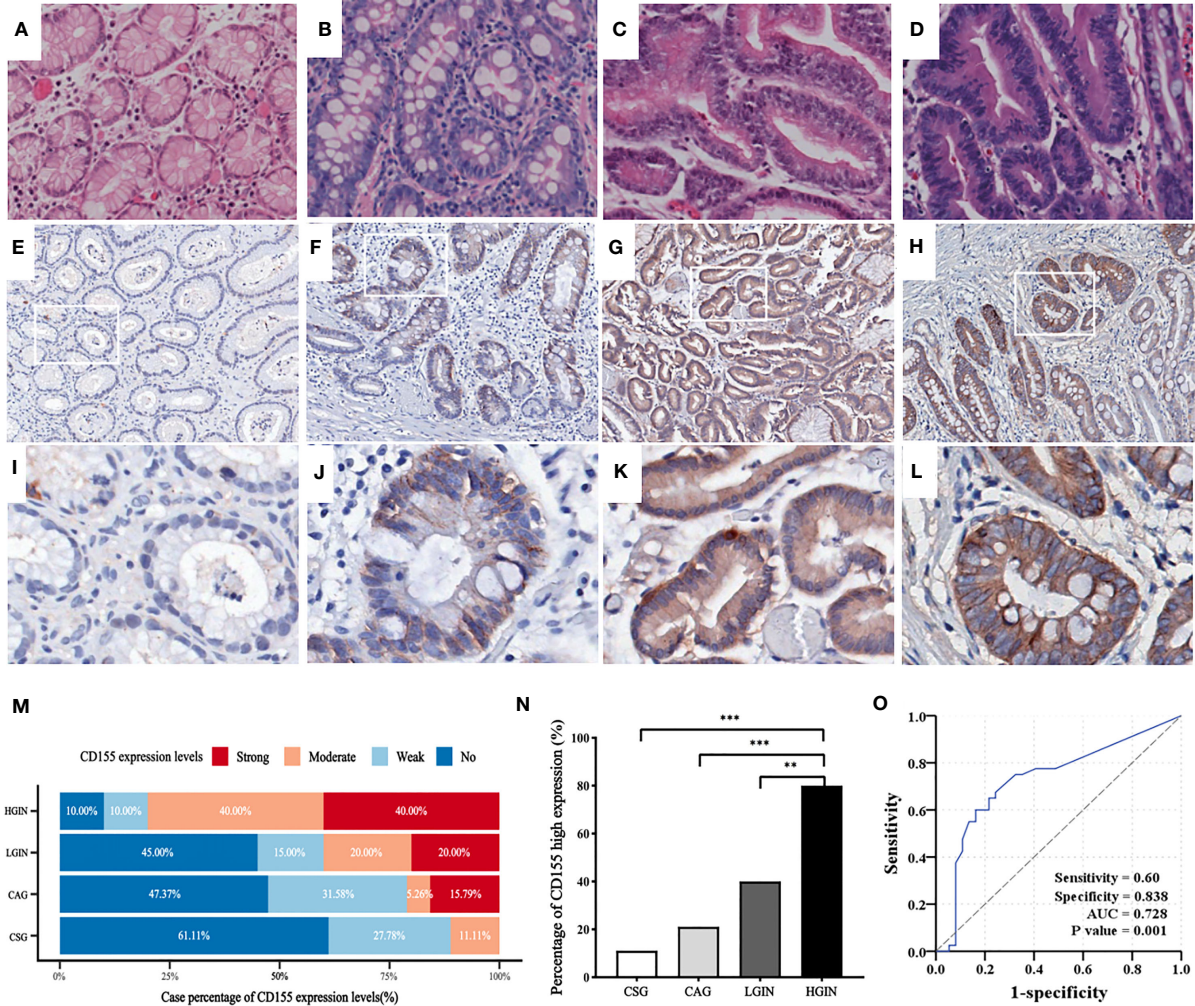
CD155 expression in distinct stages of GAC development. **(A–D)** HE staining showing CSG, CAG, LGIN and HGIN, respectively (magnification x200). **(E–L)** Representative images of CD155 IHC staining in above stages, respectively (upper: magnification x50, lower: magnification x200). **(M)** CD155 expression levels in different pathological stages. As the development of pathological stage, CD155 expression has an increasing trend in moderate and strong expression groups, and decreasing trend in no and weak groups. **(N)** Moderate and strong expression were defined as high expression. The proportion of high expression of CD155 has significant difference in four progressing stages (11.1%, 21.1%, 40% and 80% respectively, *P <*0.0001). Spearman correlation demonstrated that CD155 high expression was positively correlated with the pathological evolution of GAC (r = 0.521, *P <*0.0001). **(O)** ROC curve showed predictive power of CD155 expression in process from gastritis to intraepithelial neoplasia by H-score. **P < 0.01, ***P < 0.001. CSG, chronic superficial gastritis; CAG, chronic atrophic gastritis; LGIN, low-grade intraepithelial neoplasia; HGIN, high-grade intraepithelial neoplasia.

The representative images of HE-staining for each precancerous lesion are shown in **Figures 1A–D**. IHC showed that CD155 expression was mainly localized in the mucosal cells with varying levels (**Figures 1E–L**). As the disease risk heading to GAC increased, the patient cases with strong CD155 expression gradually rose from 0 (0%) in the CSG to 8 (40%) in the HGIN, while the cases with no CD155 expression gradually decreased from 11 (55%) in the CSG to 2 (10%) in the HGIN (**Table S1**). The findings revealed that the differences in CD155 expression levels and the proportion of CD155 high expression groups were statistically significant when comparing each pathological stage (χ^2^ = 28.906, *P* = 0.001 and χ^2^ = 22.606, *P* < 0.0001, respectively), with the ratio of CD155 high expression in the HGIN group being markedly higher than that in the CSG, CAG and LGIN groups (*P* < 0.05; **Figures 1M, N**). Additionally, Spearman analysis displayed a positive correlation between CD155 high expression and progression of gastric precancerous lesions, which tended to progress as the percentage of CD155 expression increased (r = 0.521, *P* < 0.0001) (**Figure 1N**; **Table S1**). We next evaluated the diagnostic significance of CD155 in gastritis and intraepithelial neoplasia via ROC analysis. The cutoff value of H-score was 115 with an area under the curve (AUC) of 0.728 (95% CI: 0.610-0.846, *P* = 0.001), sensitivity of 60.0%, and specificity of 83.8% (**Figure 1O**).

### 3.2 Association of CD155 with gastric cancer progression in TCGA-STAD dataset

**Figure 2:**
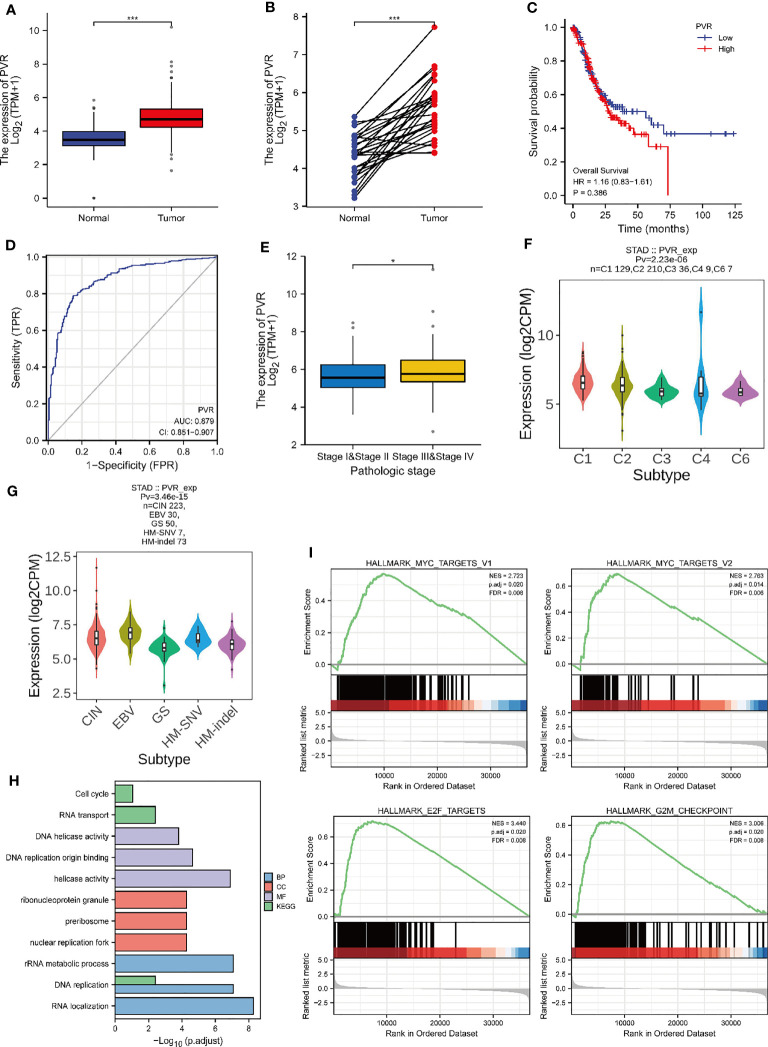
Relationship between CD155 mRNA expression and gastric cancer progression. The expression level of CD155 in non-paired tumors **(A)** and paired tumors **(B)** in comparison with normal tissues. **(C)** The overall survival of CD155 in the TCGA database. **(D)** ROC curve analysis of CD155 diagnosis. **(E)** The CD155 mRNA expression Level in STAD patients with different pathologic stages. **(F)** CD155 expression level in five immune subtypes in STAD: C1 (wound healing), C2 (IFN-gamma dominant), C3 (inflammatory), C4 (lymphocyte depleted), and C6 (TGF-b dominant). **(G)** CD155 expression in five molecular subtypes of STAD: CIN, Chromosome Instable; EBV, Epstein Barr virus; GS, Genetic Stable; HM-SNV, Hypermutated-SNV; HM-indel, Hypermutated-indel. **(H)** GO and KEGG functional enrichment analysis of CD155. **(I)** Gene set enrichment analysis. *P < 0.05; ***P < 0.001.

To investigate the role of CD155 in GC, we first analyzed the mRNA expression level of CD155 in the TCGA-STAD database. CD155 was significantly upregulated in STAD tumor tissues (**Figures 2A, B**). Next, Kaplan-Meier survival curve was performed to explore the relationship between CD155 and prognosis of GC. Evidently, CD155 overexpression was related to poor prognosis in GC patients (**Figure 2C**). For evaluating the diagnostic efficacy of CD155 in GC, we conducted ROC curve and yielded an AUC value of 0.87, representing good diagnostic performance (**Figure 2D**). Moreover, we analyzed the CD155 expression level in different pathological stages and molecular typing. The outcomes showed that CD155 expression was associated with GC patient stage (**Figure 2E**). Meanwhile, there were significant differences in the CD155 expression among different molecular subtypes and immune subtypes (**Figures 2F, G**), indicating its role in the formation of the TME. To characterize the specific functions of CD155 in GC, GO and KEGG enrichment analyses were performed. The analysis results were mainly enriched in DNA replication, RNA transport, cell cycle, and so on (**Figure 2H**). This biological process is most closely associated with cell proliferation. At the same time, GSEA results showed enrichment for several cell-cycle related pathways, such as MYC, E2F targets, and G2M checkpoint pathways (**Figure 2I**). Thus, we hypothesized that there may be certain link between the expression of CD155 and the proliferative capacity of tumor cells.

### 3.3 Correlation of CD155 expression with clinicopathological characteristics and prognosis of 268 GAC samples

**Figure 3:**
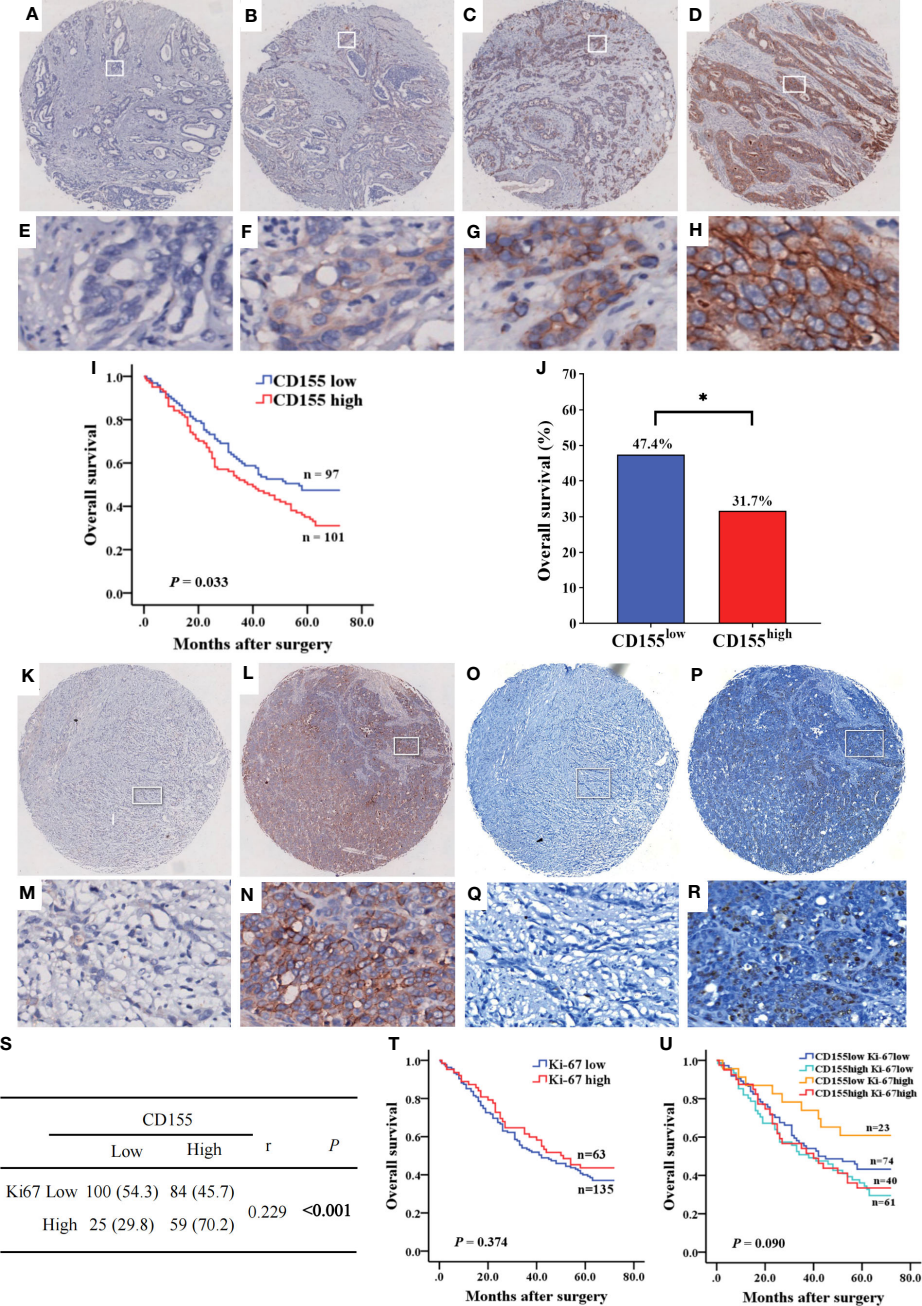
The expression of CD155 in GAC tissue and its relationship with prognosis of patients. **(A–H)** Levels of CD155 expression by IHC staining was graded as no, weak, moderate, and strong respectively (above, magnification x25; below, magnification x200). **(I)** Kaplan-Meier survival curve showed significant difference between low and high CD155 expression (*P* = 0.033). **(J)** Overall survival rate was significantly higher in low CD155 expression than high (47.4% vs. 31.7%, *P* = 0.023). *P < 0.05. **(K–R)** Identification of CD155 and Ki67 expression in the same GAC samples. **(K–N)** IHC staining of CD155. **(O–R)** IHC staining of Ki67. **(S)** Correlation between CD155 and Ki67 expression by Spearman analysis. **(T)** Kaplan-Meier survival curve according to low and high levels of Ki67. **(U)** Kaplan-Meier survival curve according to the combination of CD155 and Ki67 expression. *P < 0.05.

Subsequently, we analyzed CD155 expression in 268 GAC tissues by IHC, showing that CD155 was mainly localized in membrane and cytoplasm of tumor cells (**Figures 3A–H**). The statistical analysis of the relationship between CD155 expression and clinicopathological features revealed that CD155 expression was significantly correlated with the tumor volume (r = 0.183, *P* = 0.003), tumor stage (r = 0.128, *P* = 0.037) and lymph node metastasis (r = 0.151, *P* = 0.013, **Table 1**), which also indicated that CD155 might be involved in tumor progression in gastric cancer. Kaplan-Meier survival curves showed that patients with high CD155 expression had a worse prognosis (log-rank, *P* = 0.033) (**Figure 3I**). While overall survival was higher in patients in the CD155 low expression group (χ^2 =^ 5.134, *P* = 0.023, **Figure 3J**). For examining the relationship between CD155 expression and gastric cancer cell proliferation, we evaluated the expression of CD155 and Ki67. The analysis showed that CD155 expression was in accordance with Ki67 (**Figures 3K–R**). According to the correlation analysis, CD155 expression was positively correlated with Ki67 in GAC (r = 0.229, *P* < 0.001, **Figure 3S**). We then counted four subsets based on the staining results of CD155 and Ki67, and analyzed the prognostic differences between the subgroups. K-M curves showed no difference between high or low Ki67 expression and prognosis (**Figure 3T**). There were no significant differences in overall survival among the four subgroups, but the patients with low CD155 together with high Ki67 expression had a tendency to have a better prognosis (**Figure 3U**).

**Table 1:** CD155 expression and clinicopathological characteristics.

Clinical or pathological characteristics	Total	CD155		*P*
		Low	High	
All cases	268	125 (46.6)	143 (53.4)	
Sex				0.381
Male	210	95 (45.2)	115 (54.8)	
Female	58	30 (51.7)	28 (48.3)	
Age (years)				0.531
<70	164	74 (45.1)	90 (54.9)	
≥70	104	51 (49.0)	53 (51.0)	
Tumor volume (cm^3^)				0.003
<5	186	98 (52.7)	88 (47.3)	
≥5	82	27 (32.9)	55 (67.1)	
Tumor differentiation				0.973
Well	6	3 (50.0)	3 (50.0)	
Moderate	121	57 (47.1)	64 (52.9)	
Poor	141	65 (46.1)	76 (53.9)	
Tumor stage				0.046
0	11	4 (36.4)	7 (63.6)	
1	32	21 (65.6)	11 (34.4)	
2	66	36 (54.5)	30 (45.5)	
3	123	48 (39.0)	75 (61.0)	
4	36	16 (44.4)	20 (55.6)	
Tumor depth				0.048
T1	36	20 (55.6)	16 (44.4)	
T2	34	21 (61.8)	13 (38.2)	
T3	169	68 (40.2)	101 (59.8)	
T4	29	16 (55.2)	13 (44.8)	
Lymph node involvement				0.002
N0	85	45 (52.9)	40 (47.1)	
N1	62	38 (61.3)	24 (38.7)	
N2	56	16 (28.6)	40 (71.4)	
N3	65	26 (40.0)	39 (60.0)	
Metastasis				0.439
M0	238	113 (47.5)	125 (52.5)	
M1	30	12 (40.0)	18 (60.0)	

### 3.4 Association of CD155 expression with infiltrating immune cells in TCGA-STAD dataset

**Figure 4:**
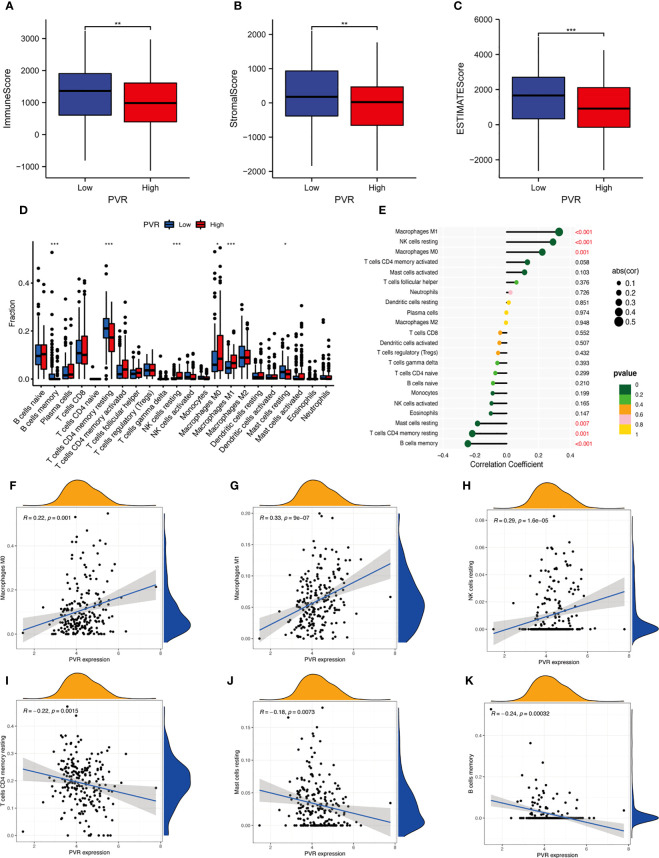
Correlation between CD155 and tumor immune infiltration. **(A–C)** Box plots show the immune score **(A)**, stromal score **(B)**, and ESTIMATE score **(C)** between low- and high-CD155 groups. **(D)** Relative infiltrating proportion of immune cells in high- and low-CD155 groups. **(E)** Lollipop graph shows the correlation between CD155 expression and immune cells. **(F–K)** Scatter plots present the correlation between CD155 expression and immune cells. *p < 0.05; **p < 0.01; ***p < 0.001.

For exploring the effect of CD155 on the TME, we first determined whether CD155 expression was related to tumor immune cell infiltration in STAD using the bioinformatic methods. We found that the stromal, immune, and ESTIMATE score for the TME of STAD patients were lower in the high-CD155 group (**Figures 4A–C**). In addition, CIBERSORT algorithm was used to assess the influence of CD155 expression level on immune cell infiltration. The results indicated a significant positive correlation between CD155 expression and specific immune cells, such as M0 macrophages, M1 macrophages, and resting NK cells. The converse was observed with resting CD4 memory T cells, resting mast cells, and memory B cells (**Figures 4D–K**). Thus, we speculated that CD155 plays critical and complicated roles with TME cells.

### 3.5 Infiltration level of TME cells in GAC and relationship with prognosis

**Figure 5:**
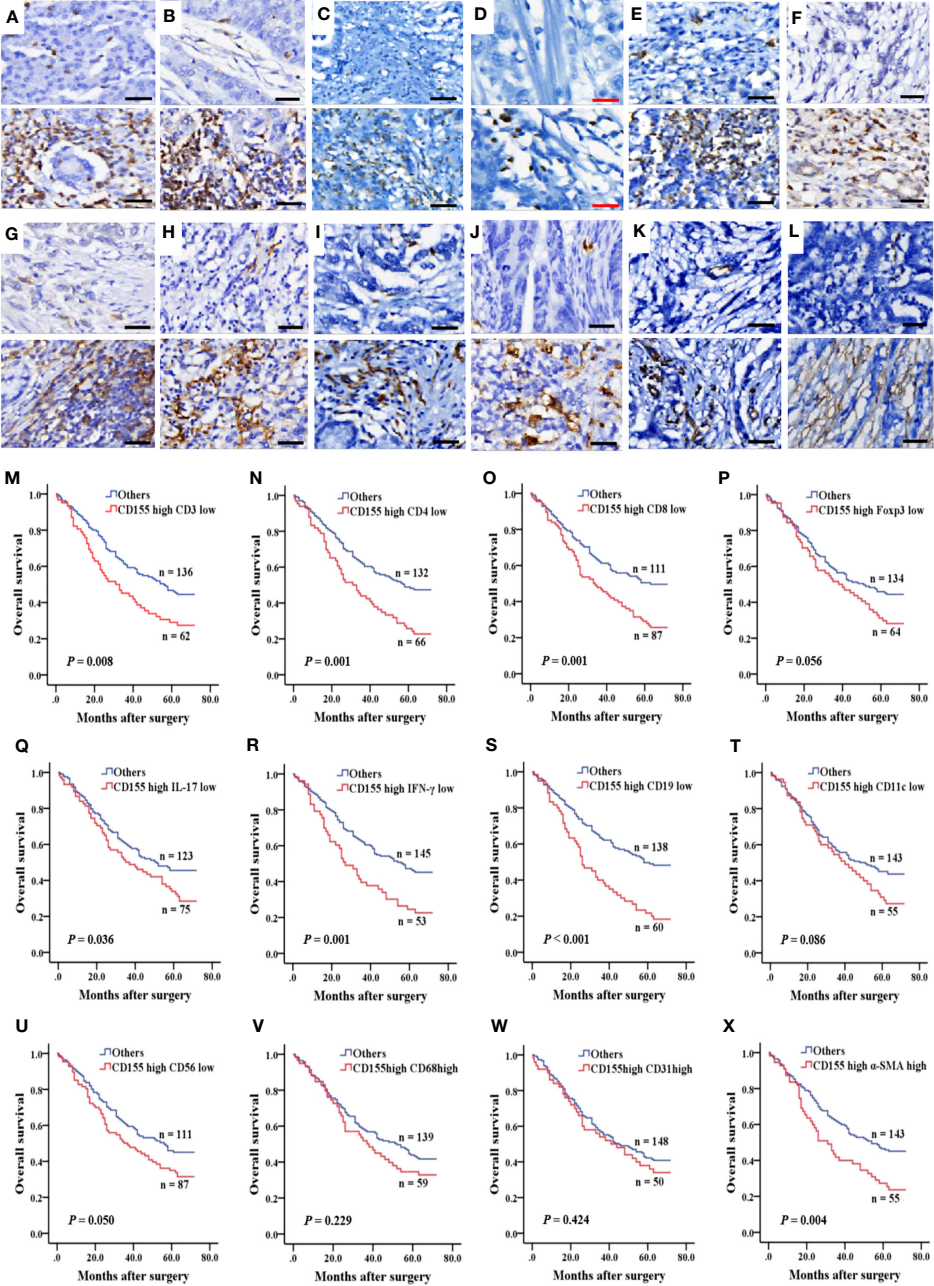
Immune and stromal cell infiltration and prognosis predicted by combined CD155. **(A–L)** IHC was performed to measure the levels of immune and stromal cell markers for CD3, CD4, CD8, Foxp3, IL-17, IFN-γ, CD19, CD11c, CD56, CD68, CD31 and α-SMA in 268 GAC samples. Molecules levels of TME cells was graded as low and high levels (upper: low levels, lower: high levels). Black bar: 50 µm, red bar: 25 µm. **(M–X)** Kaplan-Meier survival curve according to combination of expression of CD155 and TME cells markers.

To evaluate the relationship between CD155 expression and immune or stromal cell infiltration in TME and prognosis in GAC patients, we analyzed the expression of TME cellular markers, including CD3, CD4, CD8, Foxp3, IL-17, IFN-γ, CD19, CD11c, CD56, CD68, CD31, and α-SMA (**Figures 5A–L**), of which the levels were found to correlate with some clinicopathological features in GAC (**Tables S2**–**S5**). The difference in survival between the low and high expression of TME cell markers was presented by Kaplan-Meier survival curves (**Figure S1**). Then, the correlation between CD155 expression and TME landscape was evaluated. The positive correlation was only observed between CD155 and CD68 (r = 0.155, *P* = 0.011) and between CD155 and CD31 (r = 0.136, *P* = 0.026, **Table 2**). We further combined CD155 expression with these markers to assess prognostic differences (**Figures 5M–X**). Patients with high CD155 expression combined with low CD3, CD4, CD8, IL-17, IFN-γ or CD19 expression were associated with poorer overall survival (log-rank, *P* = 0.008, *P* = 0.001, *P* = 0.001, *P* = 0.036, *P* = 0.001, *P* < 0.001, respectively). The overall survival was shorter in patients with high expression of both CD155 and α-SMA (*P* = 0.004).

**Table 2:** Correlations between CD155 expression and markers levels of TME cells.

	CD155		r	*P*
	Low	High		
CD3 Low	78	89	0.002	0.978
High	47	54		
CD4 Low	78	92	-0.02	0.744
High	47	51		
CD8 Low	92	118	-0.108	0.077
High	33	25		
Foxp3 Low	65	83	-0.061	0.323
High	60	60		
IL-17 Low	81	107	-0.109	0.074
High	44	36		
IFN-γ Low	50	72	-0.104	0.090
High	75	71		
CD19 Low	73	82	0.011	0.862
High	52	61		
CD11c Low	66	69	0.045	0.459
High	59	74		
CD56 Low	112	124	0.044	0.469
High	13	19		
CD68 Low	71	59	0.155	0.011
High	54	84		
CD31 Low	79	71	0.136	0.026
High	46	72		
α-SMA Low	44	55	-0.034	0.583
High	81	88		

### 3.6 Univariate and multivariate Cox regression analysis

Univariate Cox proportional Hazard regression analysis for all variables showed that tumor volume, tumor stage, depth of tumor infiltration, lymph node involvement, distant metastasis, CD155, CD4, CD8, IFN-γ, CD19, α-SMA, as well as CD155 and TME molecules co-expression patterns for CD155/CD3, CD155/CD4, CD155/CD8, CD155/IL-17, CD155/IFN-γ, CD155/CD19, and CD155/α-SMA were factors affecting postoperative survival in GAC patients (**Table 3**). Meanwhile, Multivariate Cox regression analysis of survival related to co-expression of CD155 and TME biomarkers adjusted for clinicopathologic features showed that co-expression model of CD155/CD3, CD155/CD4 and CD155/CD19 served as independent factors for overall survival in GAC patients (**Table 4**).

**Table 3:** Univariate Cox regression analysis of survival.

Variables	Univariate analysis	*P*
	HR (95% CI)	
Sex (female vs. male)	0.901 (0.583-1.391)	0.638
Age (<70 years vs. ≥70 years)	1.203 (0.839-1.724)	0.315
Tumor volume (<5 cm³ vs. ≥5 cm³)	1.829 (1.267-2.640)	0.001
Tumor differentiation (well, moderate vs. poor)	1.030 (0.745-1.424)	0.859
Tumor stage (0, 1, 2, 3 vs. 4)	2.062 (1.661-2.560)	<0.001
Tumor depth (T1, T2, T3 vs. T4)	1.737 (1.383-2.181)	<0.001
Lymph node involvement (N0, N1, N2 vs. N3)	1.797 (1.527-2.115)	<0.001
Metastasis (M0 vs. M1)	2.118 (1.278-3.509)	0.004
CD155 (low vs. high)	1.476 (1.027-2.121)	0.035
CD155/CD3 (others vs. high/low)	1.643 (1.134-2.380)	0.009
CD155/CD4 (others vs. high/low)	1.863 (1.295-2.679)	0.001
CD155/CD8 (others vs. high/low)	1.788 (1.247-2.562)	0.002
CD155/Foxp3 (others vs. high/low)	1.425 (0.986-2.060)	0.060
CD155/IL-17 (others vs. high/low)	1.465 (1.021-2.101)	0.038
CD155/IFN-γ (others vs. high/low)	1.831 (1.253-2.674)	0.002
CD155/CD19 (others vs. high/low)	2.145 (1.486-3.095)	<0.001
CD155/CD11c (others vs. high/low)	1.390 (0.951-2.033)	0.089
CD155/CD56 (others vs. high/low)	1.425 (0.996-2.040)	0.053
CD155/CD68 (others vs. high/low)	1.262 (0.861-1.849)	0.234
CD155/CD31 (others vs. high/low)	1.176 (0.788-1.756)	0.427
CD155/α-SMA (others vs. high/low)	1.716 (1.178-2.500)	0.005

**Table 4:** Multivariate Cox regression analysis of survival related to coexpression of CD155 and TME biomarkers adjusted for clinicopathologic features^1^.

Variables	Multivariate analysis	*P*
	HR (95% CI)	
Clinicopathologic features + CD155	1.160 (0.795-1.694)	0.442
Clinicopathologic features + CD155/CD3	1.500 (1.028-2.188)	0.036
Clinicopathologic features + CD155/CD4	1.488 (1.019-2.173)	0.040
Clinicopathologic features + CD155/CD8	1.378 (0.945-2.007)	0.096
Clinicopathologic features + CD155/Foxp3	1.429 (0.985-2.072)	0.060
Clinicopathologic features + CD155/IL-17	1.208 (0.825-1.767)	0.331
Clinicopathologic features + CD155/IFN-γ	1.337 (0.908-1.970)	0.141
Clinicopathologic features + CD155/CD19	1.565 (1.072-2.286)	0.020
Clinicopathologic features + CD155/CD11c	1.321 (0.896-1.948)	0.160
Clinicopathologic features + CD155/CD56	0.983 (0.673-1.436)	0.929
Clinicopathologic features + CD155/CD68	1.009 (0.683-1.491)	0.964
Clinicopathologic features + CD155/CD31	0.946 (0.630-1.420)	0.789
Clinicopathologic features + CD155/α-SMA	1.344 (0.909-1.988)	0.138

^1^Clinicopathologic features include tumor volume, tumor stage, tumor depth, lymph node involvement and metastasis (Statistically significant in **Table 3**).

## 4 Discussion

Co-inhibitory receptor molecule TIGIT is a promising therapeutic target for tumor immunotherapy, with multiple clinical trials and preclinical trials underway. CD155 is the most important and widely expressed ligand of TIGIT. The elucidation on CD155 expression changes in GC as well as in the evolution of GC is valuable for patient screening and evaluation of clinical efficacy when using anti-TIGIT antibodies in the treatment of GC. Here, we found that CD155 expression was increased gradually with disease progression and CD155 had a differential diagnostic value on gastritis and intraepithelial neoplasia disease. CD155 expression was significantly upregulated and associated with poorer overall survival and tumor progression in GAC. Moreover, considering the relationship of TME, our findings showed that CD155 expression was positively correlated with the infiltration of CD68+ macrophages in TME and that combination of CD155 expression and levels of tumor-infiltrating T or B cells could predict the prognosis in GAC patients.

The occurrence and development of GC is a complicated biological process involving multiple factors and steps. Traditionally, the typical development model of intestinal-type GC, described by the Correa classification, is a progression from chronic gastritis, atrophic gastritis, atrophic gastritis with intestinal metaplasia to dysplasia, eventually developing into carcinoma (32). We found that the CD155 expression level was lower in gastritis and enhanced in neoplastic tissues, especially in the HGIN stage, and high CD155 expression had a marked positive correlation with the progression of the lesion. On one hand, the results indicated that CD155 has diagnostic value for lesions of different character in the stomach, which may be beneficial for the diagnosis of gastric cancer at an earlier stage. On the other hand, we speculated that CD155 may drive the evolutionary process from gastritis to gastric intraepithelial neoplasia in combination with the close relationship between CD155 and cell proliferation. Previous studies have revealed that the expression of CD155 was significantly higher in neoplastic tissues such as intestinal adenomas and high-grade cervical squamous intraepithelial lesions than in normal tissues (22, 33), consistently with our results, suggesting that CD155 might play a role in tumorigenesis. However, it is not completely clear which exact mechanism is involved. DNA damage, an important pathological process normally activated in precancerous cells, could induce CD155 expression (34, 35). It suggests that CD155 may be a stress-induced ligand, in part reflecting potential danger and malignant transformation inside the body. Meanwhile, CD155 was associated with multiple DNA and RNA biological activities analyzed in high-throughput data, which is supposed to be a potential mechanism for CD155 to promote tumor cell proliferation. Moreover, CD155 enhanced the serum- and platelet-derived growth factor- induced activation of the Ras-Raf-MEK-ERK signaling, up-regulated cyclins D2 and E, and down-regulated p27^Kip1^, shortened the period of the G0/G1 phase of the cell cycle, eventually promoting cell proliferation (36). Further studies in a variety of tumor cells have also demonstrated that CD155 can promote tumor cell proliferation by regulating cell cycle-associated proteins and cell cycle progression (16, 17, 22). Just as PD-L1 expression can exert an impact on the therapeutic efficacy of anti-PD1 antibodies, the tight association of CD155 with cell proliferation may also influence the clinical treatment efficacy of anti-TIGIT antibodies.

We showed here that CD155 was closely associated with the aggressive clinicopathological characteristics including tumor volume, tumor stage, and lymph node involvement according to the analysis of TCGA-database and 268 GAC tissue specimens. Several studies have demonstrated that upregulated expression of CD155 in cancer cells enhanced tumor proliferation, invasion, migration and distant metastasis (22, 37–39). In contrast, knockdown of CD155 in colorectal cancer cells inhibited tumor cell proliferation, invasion, and conversely induced apoptosis via AKT/Bcl-2/Bax (16). By blocking CD155, cancer cell metastasis to the lungs were inhibited (38). In agreement with our results, these findings suggested that CD155 can not only affect gastric carcinogenesis, but contribute to the progression of GC, and consequently lead to worse prognosis as well.

The function of CD155 in tumor immunomodulation has become a research hotspot, and has attracted increasing attention. CD155 combines with CD226 to enhance T/NK cell-mediated cytotoxicity and promote anti-tumor immune response, but also interacts with TIGIT and CD96 to induce tumor immune escape and promote tumor progression (11–13). Interestingly, it was shown that the binding of CD155 to TIGIT was able to dampen the immune activity of CD226 (8, 10). Thus, the relationship between CD155 and the immune microenvironment is complex. It was reported that CD155 expression on human pancreatic cancer cells might hinder the infiltration of various tumor-infiltrating lymphocytes (TILs), which was based on the negative correlation between CD155 and TILs (17). In this study, we examined the correlations between CD155 and various immune cells and stromal cells of TME in GAC at both mRNA and protein levels, respectively, to investigate whether the tumor tissues with high CD155 expression were accompanied by an immunosuppressive state or a high degree of stromal cell infiltration, and to assess the TME landscape of GAC corresponding to different levels of CD155 expression. Our results revealed an inverse correlation which at the RNA level or no correlation which at the protein level between CD155 and infiltration of T and B lymphocytes, whereas CD155 was significantly related to the expression of tumor-infiltrating macrophages (CD68+ macrophages) which at both RNA and protein levels. Hence, it can be thought that the adverse prognosis of patients with high CD155 expression is not only related to its promotion of tumor cell proliferation, invasion and metastasis, but also might be involved in its mediated tumor immunosuppression. Tumor-associated macrophages (TAMs), one of the main types of immune cells in TME, have a major role in the development of tumors. Targeting tumor-infiltrating macrophages has also become one of the principal strategies in current tumor immunotherapy (40). Apart from our finding of an expression correlation between CD155 and macrophages, CD155 related to CD68/CD163 was also observed in breast cancer tissues (21). Nevertheless, the relationship between CD155 and tumor-infiltrating macrophages remains to be illuminated. It was observed that TIGIT interacted directly with CD155 on macrophages and inhibited M1 macrophage-mediated cytotoxicity and reduced the expression of pro-inflammatory genes such as TNFα, IL-1β and IL-12 in part via the phosphorylation of SHP-1 (41). Another study has shown that cisplatin-resistant lung cancer cells can promote M2 polarization of TAMs via Src/CD155/macrophage inhibitory factor, contributing to cancer progression (42). Thus, CD155 might influence tumor progression by inhibiting function of M1 macrophages and promoting M2 polarization. However, there are few studies on the role of CD155 in relation to CD68 in malignant tumors, especially GAC. Further exploration of the interaction between CD155 and macrophages is needed in order to discover more effective therapeutic strategies for blocking tumor immunosuppression.

Notably, the role of CD155 in tumor angiogenesis is also attracting increasingly attention. There was a positive correlation between CD155 expression and vascular endothelial growth factor (VEGF) and intratumoral micro vessel density (MVD) levels in human pancreatic cancer and cholangiocarcinoma, respectively, suggesting that CD155 contributes to angiogenesis (17, 19). Conversely, knockdown of CD155 in human umbilical vein endothelial cells inhibited the VEGF-induced capillary-like network formation (43). Together, these results suggested that CD155 could regulate VEGF-induced angiogenesis. Our findings also showed that CD155 was correlated with CD31, suggesting that CD155 may also participate in facilitating the angiogenesis of GAC, which accelerated the progression of GAC and led to unfavorable prognosis ultimately. In addition, the present study showed that the patients with high CD155 expression and low expression of CD3, CD4, CD8, IL-17, IFN-γ or CD19, as well as those with high CD155 and α-SMA expression, had a poorer prognosis. Studies on the combined CD155 expression and TME cell infiltration in relation to the prognosis of GAC patients have not been reported. Here, we revealed inconsistent results in the relationship between CD155 and T or B cells in gastric cancer based on the database analysis and clinical tissue assays, indicating that post-transcriptional modifications of CD155 might be involved. Our results illustrated the dismal prognosis of GAC patients with high-CD155 along with a decreased level of tumor-infiltrating T/B lymphocytes, indicating that the expression level of CD155 and the infiltration degree of T or B lymphocyte could be a combined indicator for the prognosis of patients, which provides a potential marker for the subsequent treatment with anti-TIGIT antibodies. Among stromal cells in TME, Cancer-associated fibroblasts (CAFs) are the most abundant and have a critical role in cancer progression (44). The worse prognosis of patients with high expression of both CD155 and α-SMA might be related to the factor that CAFs can inhibit the killing activity of NK cells through CD155, aggravating tumor immunosuppression (45). Altogether, CD155 may in part promote the suppressive effects of TME in GAC patients. Moreover, CD155 expression and levels of tumor-infiltrating T or B cells could be combined to predict the prognosis in patients with GAC.

In summary, our study showed CD155 expression was gradually increased with the pathological evolution of gastritis to intraepithelial neoplasia. High CD155 expression was associated with tumor progression and worse prognosis in GAC patients. The levels of CD155 expression combined with multiple TME cells infiltration can serve as an indicator of prognosis. Based on these findings, we conclude that CD155 plays an important role in the development of GAC, and is expected to become a novel target of immunotherapy in GAC patients.

## Data availability statement

The original contributions presented in the study are included in the article/**Supplementary Material**. Further inquiries can be directed to the corresponding authors.

## Ethics statement

The studies involving human participants were reviewed and approved by the Clinical Research Ethics Committee of the First Affiliated Hospital of Soochow University (approval number: 2018121). The patients/participants provided their written informed consent to participate in this study.

## Author contributions

XL: Methodology, Formal analysis, Writing – original draft. CX: Formal analysis, Writing – original draft. TG: Resources, Methodology. SZ: Data curation, Validation. QQ: Investigation, Methodology. ML: Formal analysis. ZW: Formal analysis. XZ Supervision. LG: Project administration, Resources, Supervision. LC: Conceptualization, Funding acquisition, Project administration, Writing – review & editing. All authors contributed to the article and approved the submitted version.
