# Supplementary figures and images for: Clinical significance of CD155 expression and correlation with cellular components of tumor microenvironment in gastric adenocarcinoma
Source: Front Immunol. 2023 Jun 27;14:1173524. doi: 10.3389/fimmu.2023.1173524 (PMC10333512; doi:10.3389/fimmu.2023.1173524)

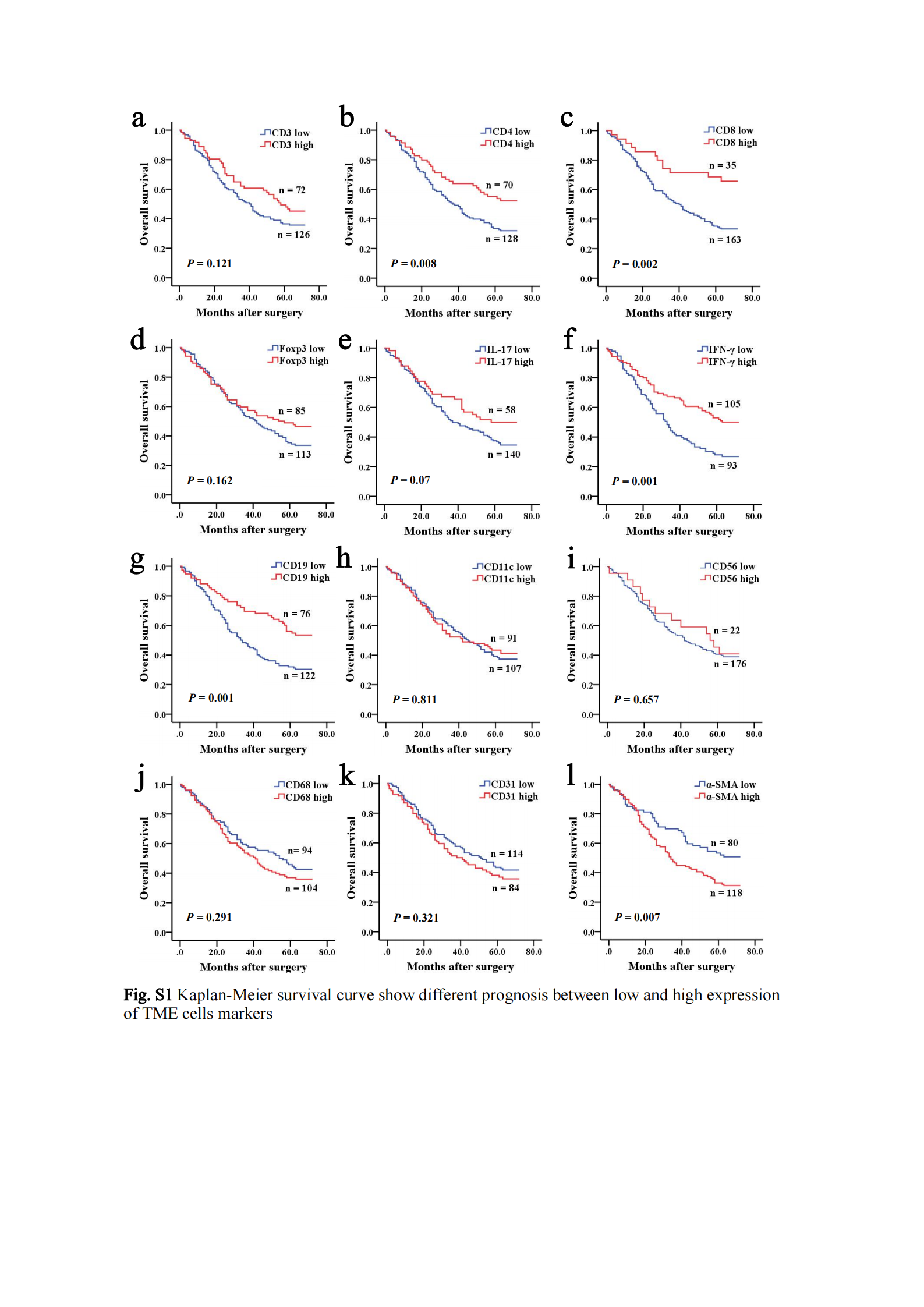

Supplement: Supplementary file 1 [file Image_1.tif]
