# Supplementary material for: Clinical significance of CD155 expression and correlation with cellular components of tumor microenvironment in gastric adenocarcinoma
Source: Front Immunol. 2023 Jun 27;14:1173524. doi: 10.3389/fimmu.2023.1173524 (PMC10333512; doi:10.3389/fimmu.2023.1173524)
Supplement: Supplementary file 2 [file Table_1.docx]

**Table S1.** CD155 expression in distinct stages of GAC development.

| Pathological stages | CD155 expression | | | | Total |
| --- | --- | --- | --- | --- | --- |
|  | No | Weak | Moderate | Strong |  |
| CSG | 11 (61.11) | 5 (27.78) | 2 (11.11) | 0 (0) | 18 |
| CAG | 9 (47.37) | 6 (31.58) | 1 (5.26) | 3 (15.79) | 19 |
| LGIN | 9 (45.00) | 3 (15.00) | 4 (20.00) | 4 (20.00) | 20 |
| HGIN | 2 (10.00) | 2 (10.00) | 8 (40.00) | 8 (40.00) | 20 |

CSG, chronic superficial gastritis; CAG, chronic atrophic gastritis; LGIN, low-grade intraepithelial neoplasia; HGIN, high-grade intraepithelial neoplasia.

**Table S2.** CD3, CD4, CD8 and clinical or pathological characteristics

| **Clinical or pathological characteristics** | **Total** | **CD3** | | ***P*** | **CD4** | | ***P*** | **CD8** | | ***P*** |
| --- | --- | --- | --- | --- | --- | --- | --- | --- | --- | --- |
|  |  | **Low** | **High** |  | **Low** | **High** |  | **Low** | **High** |  |
| **All cases** | 268 | 167 (62.3) | 101 (37.7) |  | 170 (63.4) | 98 (36.6) |  | 210 (78.4) | 58 (21.6) |  |
| **Sex** |  |  |  | 0.382 |  |  | 0.710 |  |  | 0.214 |
| Male | 210 | 128 (61.0) | 82 (39.0) |  | 132 (62.9) | 78 (37.1) |  | 168 (80.0) | 42 (20.0) |  |
| Female | 58 | 39 (67.2) | 19 (32.8) |  | 38 (65.5) | 20 (34.5) |  | 42 (72.4) | 16 (27.6) |  |
| **Age (yrs)** |  |  |  | 0.179 |  |  | 0.597 |  |  | 0.881 |
| <70 | 164 | 97 (59.1) | 67 (40.9) |  | 102 (62.2) | 62 (37.8) |  | 129 (78.7) | 35 (21.3) |  |
| ≥70 | 104 | 70 (67.3) | 34 (32.7) |  | 68 (65.4) | 36 (34.6) |  | 81 (77.9) | 23 (22.1) |  |
| **Tumor volume (cm^3^)** |  |  |  | 0.566 |  |  | 0.269 |  |  | 0.935 |
| <5 | 186 | 118 (63.4) | 68 (36.6) |  | 122 (65.6) | 64 (34.4) |  | 146 (78.5) | 40 (21.5) |  |
| ≥5 | 82 | 49 (59.8) | 33 (40.2) |  | 48 (58.5) | 34 (41.5) |  | 64 (78.0) | 18 (22.0) |  |
| **Tumor differentiation** |  |  |  | 0.332 |  |  | **0.030** |  |  | 0.081 |
| Well | 6 | 3 (50.0) | 3 (50.0) |  | 3 (50.0) | 3 (50.0) |  | 6 (100.0) | 0 (0.0) |  |
| Moderate | 121 | 81 (66.9) | 40 (33.1) |  | 87 (71.9) | 34 (28.1) |  | 99 (81.8) | 22 (18.2) |  |
| Poor | 141 | 83 (58.9) | 58 (41.1) |  | 80 (56.7) | 61 (43.3) |  | 105 (74.5) | 36 (25.5) |  |
| **Tumor stage** |  |  |  | 0.379 |  |  | 0.924 |  |  | 0.796 |
| 0 | 11 | 9 (81.8) | 2 (18.2) |  | 7 (63.6) | 4 (36.4) |  | 8 (72.7) | 3 (27.3) |  |
| Ⅰ | 32 | 19 (59.4) | 13 (40.6) |  | 21 (65.6) | 11 (34.4) |  | 23 (71.9) | 9 (28.1) |  |
| Ⅱ | 66 | 39 (59.1) | 27 (40.9) |  | 40 (60.6) | 26 (39.4) |  | 51 (77.3) | 15 (22.7) |  |
| Ⅲ | 123 | 74 (60.2) | 49 (39.8) |  | 77 (62.6) | 46 (37.4) |  | 98 (79.7) | 25 (20.3) |  |
| Ⅳ | 36 | 26 (72.2) | 10 (27.8) |  | 25 (69.4) | 11 (30.6) |  | 30 (83.3) | 6 (16.7) |  |
| **Tumor depth** |  |  |  | 0.597 |  |  | 0.628 |  |  | 0.499 |
| T1 | 36 | 22 (61.1) | 14 (38.9) |  | 21 (58.3) | 15 (41.7) |  | 29 (80.6) | 7 (19.4) |  |
| T2 | 34 | 19 (55.9) | 15 (44.1) |  | 20 (58.8) | 14 (41.2) |  | 24 (70.6) | 10 (29.4) |  |
| T3 | 169 | 105 (62.1) | 64 (37.9) |  | 108 (63.9) | 61 (36.1) |  | 132 (78.1) | 37 (21.9) |  |
| T4 | 29 | 21 (72.4) | 8 (27.6) |  | 21 (72.4) | 8 (27.6) |  | 25 (86.2) | 4 (13.8) |  |
| **Lymph node involvement** |  |  |  | 0.914 |  |  | 0.805 |  |  | 0.317 |
| N0 | 85 | 55 (64.7) | 30 (35.3) |  | 56 (65.9) | 29 (34.1) |  | 62 (72.9) | 23 (27.1) |  |
| N1 | 62 | 38 (61.3) | 24 (38.7) |  | 37 (59.7) | 25 (40.3) |  | 50 (80.6) | 12 (19.4) |  |
| N2 | 56 | 33 (58.9) | 23 (41.1) |  | 34 (60.7) | 22 (39.3) |  | 48 (85.7) | 8 (14.3) |  |
| N3 | 65 | 41 (63.1) | 24 (36.9) |  | 43 (66.2) | 22 (33.8) |  | 50 (76.9) | 15 (23.1) |  |
| **Metastasis** |  |  |  | 0.903 |  |  | 0.990 |  |  | 0.483 |
| M0 | 238 | 148(62.2) | 90 (37.8) |  | 151 (63.4) | 87 (36.6) |  | 185(77.7) | 53 (22.3) |  |
| M1 | 30 | 19 (63.3) | 11 (36.7) |  | 19 (63.3) | 11 (36.7) |  | 25 (83.3) | 5 (16.7) |  |
| **Death** |  |  |  | 0.161 |  |  | **0.004** |  |  | **<0.001** |
| No | 78 | 45 (57.7) | 33 (42.3) |  | 41 (52.6) | 37 (47.4) |  | 55 (70.5) | 23 (29.5) |  |
| Yes | 120 | 81 (67.5) | 39 (32.5) |  | 87 (72.5) | 33 (27.5) |  | 108 (90.0) | 12 (10.0) |  |

**Table S3.** Foxp3, IL-17, IFN-γ and clinical or pathological characteristics

| **Clinical or pathological characteristics** | **Total** | **Foxp3** | | ***P*** | **IL-17** | | ***P*** | **IFN-γ** | | ***P*** |
| --- | --- | --- | --- | --- | --- | --- | --- | --- | --- | --- |
|  |  | **Low** | **High** |  | **Low** | **High** |  | **Low** | **High** |  |
| **All cases** | 268 | 148 (55.2) | 120 (44.8) |  | 188 (70.1) | 80 (29.9) |  | 122 (45.5) | 146 (54.5) |  |
| **Sex** |  |  |  | 0.759 |  |  | 0.824 |  |  | 0.676 |
| Male | 210 | 117 (55.7) | 93 (44.3) |  | 148 (70.5) | 62 (29.5) |  | 97 (46.2) | 113 (53.8) |  |
| Female | 58 | 31 (53.4) | 27 (46.6) |  | 40 (69.0) | 18 (31.0) |  | 25 (43.1) | 33 (56.9) |  |
| **Age (yrs)** |  |  |  | 0.250 |  |  | 0.103 |  |  | 0.869 |
| <70 | 164 | 86 (52.4) | 78 (47.6) |  | 121 (73.8) | 43 (26.2) |  | 74 (45.1) | 90 (54.9) |  |
| ≥70 | 104 | 62 (59.6) | 42 (40.4) |  | 67 (64.4) | 37 (35.6) |  | 48 (46.2) | 56 (53.8) |  |
| **Tumor volume (cm^3^)** |  |  |  | 0.647 |  |  | 0.669 |  |  | 0.214 |
| <5 | 186 | 101 (54.3) | 85 (45.7) |  | 129 (69.4) | 57 (30.6) |  | 80 (43.0) | 106 (57.0) |  |
| ≥5 | 82 | 47 (57.3) | 35 (42.7) |  | 59 (72.0) | 23 (28.0) |  | 42 (51.2) | 40 (48.8) |  |
| **Tumor differentiation** |  |  |  | 0.124 |  |  | 0.358 |  |  | **0.021** |
| Well | 6 | 1 (16.7) | 5 (83.3) |  | 5 (83.3) | 1 (16.7) |  | 5 (83.3) | 1 (16.7) |  |
| Moderate | 121 | 70 (57.9) | 51 (42.1) |  | 80 (66.1) | 41 (33.9) |  | 62 (51.2) | 59 (48.8) |  |
| Poor | 141 | 77 (54.6) | 64 (45.4) |  | 103 (73.0) | 38 (27.0) |  | 55 (39.0) | 86 (61.0) |  |
| **Tumor stage** |  |  |  | 0.928 |  |  | 0.410 |  |  | **0.001** |
| 0 | 11 | 6 (54.5) | 5 (45.5) |  | 8 (72.7) | 3 (27.3) |  | 0 (0.0) | 11 (100.0) |  |
| Ⅰ | 32 | 16 (50.0) | 16 (50.0) |  | 18 (56.3) | 14 (43.8) |  | 11 (34.4) | 21 (65.6) |  |
| Ⅱ | 66 | 36 (54.5) | 30 (45.5) |  | 46 (69.7) | 20 (30.3) |  | 25 (37.9) | 41 (62.1) |  |
| Ⅲ | 123 | 68 (55.3) | 55 (44.7) |  | 88 (71.5) | 35 (28.5) |  | 63 (51.2) | 60 (48.8) |  |
| Ⅳ | 36 | 22 (61.1) | 14 (38.9) |  | 28 (77.8) | 8 (22.2) |  | 23 (63.9) | 13 (36.1) |  |
| **Tumor depth** |  |  |  | 0.991 |  |  | 0.499 |  |  | **< 0.001** |
| T1 | 36 | 19 (52.8) | 17 (47.2) |  | 23 (63.9) | 13 (36.1) |  | 6 (16.7) | 30 (83.3) |  |
| T2 | 34 | 19 (55.9) | 15 (44.1) |  | 27 (79.4) | 7 (20.6) |  | 11 (32.4) | 23 (67.6) |  |
| T3 | 169 | 94 (55.6) | 75 (44.4) |  | 119 (70.4) | 50 (29.6) |  | 90 (53.3) | 79 (46.7) |  |
| T4 | 29 | 16 (55.2) | 13 (44.8) |  | 19 (65.5) | 10 (34.5) |  | 15 (51.7) | 14 (48.3) |  |
| **Lymph node involvement** |  |  |  | 0.867 |  |  | 0.109 |  |  | 0.116 |
| N0 | 85 | 45 (52.9) | 40 (47.1) |  | 52 (61.2) | 33 (38.8) |  | 31 (36.5) | 54 (63.5) |  |
| N1 | 62 | 37 (59.7) | 25 (40.3) |  | 43 (69.4) | 19 (30.6) |  | 33 (53.2) | 29 (46.8) |  |
| N2 | 56 | 31 (55.4) | 25 (44.6) |  | 42 (75.0) | 14 (25.0) |  | 30 (53.6) | 26 (46.4) |  |
| N3 | 65 | 35 (53.8) | 30 (46.2) |  | 51 (78.5) | 14 (21.5) |  | 28 (43.1) | 37 (56.9) |  |
| **Metastasis** |  |  |  | 0.343 |  |  | 0.211 |  |  | **0.038** |
| M0 | 238 | 129 (54.2) | 109 (45.8) |  | 164 (68.9) | 74 (31.1) |  | 103 (43.3) | 135 (56.7) |  |
| M1 | 30 | 19 (63.3) | 11 (36.7) |  | 24 (80.0) | 6 (20.0) |  | 19 (63.3) | 11 (36.7) |  |
| **Death** |  |  |  | 0.056 |  |  | **0.049** |  |  | **0.001** |
| No | 78 | 38 (48.7) | 40 (51.3) |  | 49 (62.8) | 29 (37.2) |  | 25 (32.1) | 53 (67.9) |  |
| Yes | 120 | 75 (62.5) | 45 (37.5) |  | 91 (75.8) | 29 (24.2) |  | 68 (56.7) | 52 (43.3) |  |

**Table S4.** CD19, CD11c, CD56 and clinical or pathological characteristics

| **Clinical or pathological characteristics** | **Total** | **CD19** | | ***P*** | **CD11c** | | ***P*** | **CD56** | | ***P*** |
| --- | --- | --- | --- | --- | --- | --- | --- | --- | --- | --- |
|  |  | **Low** | **High** |  | **Low** | **High** |  | **Low** | **High** |  |
| **All cases** | 268 | 155 (57.8) | 113 (42.2) |  | 135 (50.4) | 133 (49.6) |  | 236 (88.1) | 32 (11.9) |  |
| **Sex** |  |  |  | 0.299 |  |  | **0.044** |  |  | 0.672 |
| Male | 210 | 118 (56.2) | 92 (43.8) |  | 99 (47.1) | 111 (52.9) |  | 184 (87.6) | 26 (12.4) |  |
| Female | 58 | 37 (63.8) | 21 (36.2) |  | 36 (62.1) | 22 (37.9) |  | 52 (89.7) | 6 (10.3) |  |
| **Age (yrs)** |  |  |  | **0.046** |  |  | 0.922 |  |  | 0.822 |
| <70 | 164 | 87 (53.0) | 77 (47.0) |  | 83 (50.6) | 81 (49.4) |  | 145 (88.4) | 19 (11.6) |  |
| ≥70 | 104 | 68 (65.4) | 36 (34.6) |  | 52 (50.0) | 52 (50.0) |  | 91 (87.5) | 13 (12.5) |  |
| **Tumor volume (cm^3^)** |  |  |  | **0.021** |  |  | 0.729 |  |  | 0.121 |
| <5 | 186 | 99 (53.2) | 87 (46.8) |  | 95 (51.1) | 91 (48.9) |  | 160 (86.0) | 26 (14.0) |  |
| ≥5 | 82 | 56 (68.3) | 26 (31.7) |  | 40 (48.8) | 42 (51.2) |  | 76 (92.7) | 6 (7.3) |  |
| **Tumor differentiation** |  |  |  | 0.214 |  |  | 0.654 |  |  | 0.375 |
| Well | 6 | 3 (50.0) | 3 (50.0) |  | 2 (33.3) | 4 (66.7) |  | 6 (100.0) | 0 (0.0) |  |
| Moderate | 121 | 77 (63.6) | 44 (36.4) |  | 60 (49.6) | 61 (50.4) |  | 109 (90.1) | 12 (9.9) |  |
| Poor | 141 | 75 (53.2) | 66 (46.8) |  | 73 (51.8) | 68 (48.2) |  | 121 (85.8) | 20 (11.9) |  |
| **Tumor stage** |  |  |  | **0.001** |  |  | 0.072 |  |  | **0.009** |
| 0 | 11 | 1 (9.1) | 10 (90.9) |  | 10 (90.9) | 1 (9.1) |  | 6 (54.5) | 5 (45.5) |  |
| Ⅰ | 32 | 15 (46.9) | 17 (53.1) |  | 18 (56.3) | 14 (43.8) |  | 25 (78.1) | 7 (21.9) |  |
| Ⅱ | 66 | 34 (51.5) | 32 (48.5) |  | 31 (47.0) | 35 (53.0) |  | 58 (87.9) | 8 (12.1) |  |
| Ⅲ | 123 | 80 (65.0) | 43 (35.0) |  | 60 (48.8) | 63 (51.2) |  | 114 (92.7) | 9 (7.3) |  |
| Ⅳ | 36 | 25 (69.4) | 11 (30.6) |  | 16 (44.4) | 20 (55.6) |  | 33 (91.7) | 3 (8.3) |  |
| **Tumor depth** |  |  |  | **< 0.001** |  |  | 0.078 |  |  | **0.004** |
| T1 | 36 | 8 (22.2) | 28 (77.8) |  | 25 (69.4) | 11 (30.6) |  | 27 (75.0) | 9 (25.0) |  |
| T2 | 34 | 19 (55.9) | 15 (44.1) |  | 14 (41.2) | 20 (58.8) |  | 28 (82.4) | 6 (17.6) |  |
| T3 | 169 | 110 (65.1) | 59 (34.9) |  | 81 (47.9) | 88 (52.1) |  | 152 (89.9) | 17 (10.1) |  |
| T4 | 29 | 18 (62.1) | 11 (37.9) |  | 15 (51.7) | 14 (48.3) |  | 29 (100.0) | 0 (0.0) |  |
| **Lymph node involvement** |  |  |  | 0.177 |  |  | 0.305 |  |  | **0.006** |
| N0 | 85 | 41 (48.2) | 44 (51.8) |  | 48 (56.5) | 37 (43.5) |  | 68 (80.0) | 17 (20.0) |  |
| N1 | 62 | 38 (61.3) | 24 (38.7) |  | 30 (48.4) | 32 (51.6) |  | 59 (95.2) | 3 (4.8) |  |
| N2 | 56 | 34 (60.7) | 22 (39.3) |  | 30 (53.6) | 26 (46.4) |  | 47 (83.9) | 9 (16.1) |  |
| N3 | 65 | 42 (64.6) | 23 (35.4) |  | 27 (41.5) | 38 (58.5) |  | 62 (95.4) | 3 (4.6) |  |
| **Metastasis** |  |  |  | 0.299 |  |  | 0.228 |  |  | 0.961 |
| M0 | 238 | 135 (56.7) | 103 (43.3) |  | 123 (51.7) | 115 (48.3) |  | 209 (87.8) | 29 (12.2) |  |
| M1 | 30 | 20 (66.7) | 10 (33.3) |  | 12 (40.0) | 18 (60.0) |  | 27 (90.0) | 3 (10.0) |  |
| **Death** |  |  |  | **0.001** |  |  | 0.530 |  |  | 0.877 |
| No | 78 | 37 (47.4) | 41 (52.6) |  | 40 (51.3) | 38 (48.7) |  | 69 (88.5) | 9 (11.5) |  |
| Yes | 120 | 85 (70.8) | 35 (29.2) |  | 67 (55.8) | 53 (44.2) |  | 107 (89.2) | 13 (10.8) |  |

**Table S5.** CD68, CD31, α-SMA and clinical or pathological characteristics

| **Clinical or pathological characteristics** | **Total** | **CD68** | | ***P*** | **CD31** | | ***P*** | **α-SMA** | | ***P*** |
| --- | --- | --- | --- | --- | --- | --- | --- | --- | --- | --- |
|  |  | **Low** | **High** |  | **Low** | **High** |  | **Low** | **High** |  |
| **All cases** | 268 | 130 (48.5) | 138 (51.5) |  | 150 (56.0) | 118 (44.0) |  | 99 (36.9) | 169 (63.1) |  |
| **Sex** |  |  |  | 0.797 |  |  | 0.175 |  |  | 0.292 |
| Male | 210 | 101 (48.1) | 109 (51.9) |  | 113 (53.8) | 97 (46.2) |  | 81 (38.6) | 129 (61.4) |  |
| Female | 58 | 29 (50.0) | 29 (50.0) |  | 37 (63.8) | 21 (36.2) |  | 18 (31.0) | 40 (69.0) |  |
| **Age (yrs)** |  |  |  | 0.539 |  |  | 0.144 |  |  | 0.713 |
| <70 | 164 | 82 (50.0) | 82 (50.0) |  | 86 (52.4) | 78 (47.6) |  | 62 (37.8) | 102 (62.2) |  |
| ≥70 | 104 | 48 (46.2) | 56 (53.8) |  | 64 (61.5) | 40 (38.5) |  | 37 (35.6) | 67 (64.4) |  |
| **Tumor volume (cm^3^)** |  |  |  | 0.317 |  |  | 0.978 |  |  | 0.936 |
| <5 | 186 | 94 (50.5) | 92 (49.5) |  | 104 (55.9) | 82 (44.1) |  | 69 (37.1) | 117 (62.9) |  |
| ≥5 | 82 | 36 (43.9) | 46 (56.1) |  | 46 (56.1) | 36 (43.9) |  | 30 (36.6) | 52 (63.4) |  |
| **Tumor differentiation** |  |  |  | 0.445 |  |  | 0.811 |  |  | 0.517 |
| Well | 6 | 2 (33.3) | 4 (66.7) |  | 4 (66.7) | 2 (33.3) |  | 3 (50.0) | 3 (50.0) |  |
| Moderate | 121 | 55 (45.5) | 66 (54.5) |  | 66 (54.5) | 55 (45.5) |  | 48 (39.7) | 73 (60.3) |  |
| Poor | 141 | 73 (51.8) | 68 (48.2) |  | 80 (56.7) | 61 (43.3) |  | 48 (34.0) | 93 (66.0) |  |
| **Tumor stage** |  |  |  | **< 0.001** |  |  | 0.282 |  |  | 0.134 |
| 0 | 11 | 10 (90.9) | 1 (9.1) |  | 7 (63.6) | 4 (36.4) |  | 3 (27.3) | 8 (72.7) |  |
| Ⅰ | 32 | 24 (75.0) | 8 (25.0) |  | 22 (68.8) | 10 (31.3) |  | 14 (43.8) | 18 (56.3) |  |
| Ⅱ | 66 | 29 (43.9) | 37 (56.1) |  | 40 (60.6) | 26 (39.4) |  | 25 (37.9) | 41 (62.1) |  |
| Ⅲ | 123 | 54 (43.9) | 69 (56.1) |  | 61 (49.6) | 62 (50.4) |  | 50 (40.7) | 73 (59.3) |  |
| Ⅳ | 36 | 13 (36.1) | 23 (63.9) |  | 20 (55.6) | 16 (44.4) |  | 7 (19.4) | 29 (80.6) |  |
| **Tumor depth** |  |  |  | **0.003** |  |  | 0.318 |  |  | **0.038** |
| T1 | 36 | 27 (75.0) | 9 (25.0) |  | 25 (69.4) | 11 (30.6) |  | 10 (27.8) | 26 (72.2) |  |
| T2 | 34 | 18 (52.9) | 16 (47.1) |  | 20 (58.8) | 14 (41.2) |  | 16 (47.1) | 18 (52.9) |  |
| T3 | 169 | 75 (44.4) | 94 (55.6) |  | 89 (52.7) | 80 (47.3) |  | 68 (40.2) | 101 (59.8) |  |
| T4 | 29 | 10 (34.5) | 19 (65.5) |  | 16 (55.2) | 13 (44.8) |  | 5 (17.2) | 24 (82.8) |  |
| **Lymph node involvement** |  |  |  | 0.071 |  |  | 0.096 |  |  | 0.797 |
| N0 | 85 | 51 (60.0) | 34 (40.0) |  | 56 (65.9) | 29 (34.1) |  | 34 (40.0) | 51 (60.0) |  |
| N1 | 62 | 25 (40.3) | 37 (59.7) |  | 35 (56.5) | 27 (43.5) |  | 22 (35.5) | 40 (64.5) |  |
| N2 | 56 | 24 (42.9) | 32 (57.1) |  | 29 (51.8) | 27 (48.2) |  | 18 (32.1) | 38 (67.9) |  |
| N3 | 65 | 30 (46.2) | 35 (53.8) |  | 30 (46.2) | 35 (53.8) |  | 25 (38.5) | 40 (61.5) |  |
| **Metastasis** |  |  |  | 0.169 |  |  | 0.758 |  |  | 0.101 |
| M0 | 238 | 119 (50.0) | 119 (50.0) |  | 134 (56.3) | 104 (43.7) |  | 92 (38.7) | 146 (61.3) |  |
| M1 | 30 | 11 (36.7) | 19 (63.3) |  | 16 (53.3) | 14 (46.7) |  | 7 (23.3) | 23 (76.7) |  |
| **Death** |  |  |  | 0.387 |  |  | 0.363 |  |  | **0.005** |
| No | 78 | 40(51.3) | 38(48.7) |  | 48(61.5) | 30(38.5) |  | 41 (52.6) | 37 (47.4) |  |
| Yes | 120 | 54(45.0) | 66(55.0) |  | 66(55.0) | 54(45.0) |  | 39 (32.5) | 81 (67.5) |  |

**Figure legends**

**Figure S1.** Kaplan-Meier survival curve show different prognosis between low and high TME cells markers expression.
